# Report of a Complicated Case of Dislocation of the Knee Joint, with Remarks

**Published:** 1857-02

**Authors:** V. H. Taliaferro

**Affiliations:** Atlanta, Ga.


### ARTICLE II.

*Report of a Complicated Case of Dislocation of the Knee Joint, with Remarks.* By V. H. TALIAFERRO, M. D., Atlanta, Ga.

Upon examination of the various records devoted to medical science, we find few indeed, comparatively, connected with Practical Surgery. This, no doubt, is owing to the scarcity of cases occurring to the greater number of practitioners, especially those of the country, where they are not so liable to rail road and machinery accidents as in large towns and cities. It may be owing, partly, also, to the fact, that physicians, whose minds must necessarily be divided upon the various branches of their profession, generally feel not entirely satisfied with their treatment of surgical cases.

The profession is too wont to select for record those cases alone which have been successfully treated. It is equally as important, that cases, unsuccessfully treated, should be made known to the profession, as it can be no proof that the treatment was unscientific, because unsuccessful. The physician's skill cannot turn aside the hand of destiny, or always shield from the stroke of death.

It is all-important to the physician, that there be kept a record of to-day's works, that the successor of to-morrow may have data in which to find the jewel, truth, that talisman which he may use for the amelioration of disease.

The acts, the thoughts, the ideas of the men of to-day, can have but little progressive influence on those of an after age, if not transmitted by pen or type.

Prof. Draper says: "Europe remained in a barbarous state until it obtained the means of perpetuating ideas; that is, to say, until it learned the art of writing."

This is an age when every thing must be utilitarian in its aspect, tendencies and results. So few articles have ever appeared upon medical record, in connection with injuries and diseases of the knee joint, I have thought it would not be uninteresting to the readers of your journal to devote a few of its pages to that important subject. More so, on account of their unfrequent occurrence; and, also, to elicit, if possible, the experience of the profession in relation to this subject.

Injuries of the knee joint, though of unfrequent occurrence, sometimes happen, even in the most retired sections, where the skill and inventive tact of the physician is called into action. As was truly remarked by a learned philosopher of medicine, that as much skill, as much care, and talent, and even more inventive genius, was requisite, in the treatment of a rustic's injury than in that of the city gentleman.

Man is subject to no accident which requires more prompt and efficient attention than injuries connected with the knee joint, especially its dislocation, as the time for remedy soon elapses, and marked deformity ensues. Sir Astley Cooper, in speaking of dislocation of this joint, has well remarked, "A considerable share of anatomical knowledge is requisite to detect the nature of these accidents, as well as to suggest the best means of reduction ; and it is much to be lamented that our students neglect to inform themselves sufficiently of the structure of the joints. They often dissect the muscles of the limb with great neatness and minuteness, then throw it away without any examination of the ligaments, the knowledge of which, in a surgical point of view, is of such great importance."

It will not be improper here to examine casually the anatomy of the knee joint. We have, first, the condyles of the femur, and proximal extremities of the tibia, making one of the largest and most complicated joints in the human structure. That the joint might be complete—that the space between the articular surfaces be filled—a bone was necessary in the tendons of the extensor muscles of the leg, so as to facilitate standing; also, that the joint might have great strength and firmness, the patella a sesamoid bone, with the ligamentum patellæ for its inferior, and tendons of the extensor muscles for its superior attachment, plays an important part in the office of the joint. From the size, complicity, great articular strength, and peculiarity of the adaptation of the heads of the bones, which forms the knee, it is liable to a variety of maladies, and to both internal and external injuries. It plays an important part in the locomotion of the body, in the osseous and muscular structure, and is highly worthy of notice in a surgical point of view. The surfaces of the bones, which make this joint, (inferior extremity of the femur and superior extremity of the tibia,) instead of being accurately joined together, are only placed in juxtaposition, the attachment being ligamentous and cartilaginous, the crucial ligaments and external and internal semilunar cartilages serving an important part in the union.

The articulation of this joint, having distinct cavities, with corresponding convexities, might be called double, also making it opposed in a great degree to lateral and rotatory motion. Since these motions are scarcely appreciable, the joint formed by the condyles of the femur and articular surface of tibia would be classed among the regular ginglymoid.

The tissues of the joint are numerous, and are denominated the cellular, fibrous and fibro cartilaginous. The synovial capsule of the knee joint proper is the largest in the body. Between the tendons of the patella and surface of the os femoris is a "cul de sac," forming a distinct synovial capsule. This elongation of the synovial membrane is greater under the vastus externus than the vastus internus.

The ligaments of the knee joint are ten in number, viz: The semilunar cartilages, the anterior and posterior cervical ligaments, the ligamentum mucosom, ligamenta alaria, long and short external and internal ligaments, the capsular ligament, and ligamentum patellæ. The origin, insertion, &c., of these, it is now unnecessary to give, as it would be tedious and not important to our present purpose. These ligaments serve to hold the knee joint in continuity, and prevent its extension beyond a straight line with the femur, and also to confine its movements alone to that of flexion and extension.

The knee joint is surrounded not only by its ligaments, but by an aponeurosis, formed by an expansion of the facia lata, also by the expansion of the tendons of several other muscles. To these there are joined a fibrous lamina, arising from the conjoined tendons of the vasti muscles. There is also a sub-synovial adipose tissue, situated behind the ligamentum patellæ, which fills the space behind the patella and synovial membrane. The number and extent of the ligaments and tendons connected with the articulating surface of the knee joint, the spine of the tibia entering the inferior condyloid fossa, its width of articulatory surface and the patella, renders it the strongest joint in the human system, also more liable to the most grave and serious diseases, such as Synovitis, Caries of the bone, Exostosis, Arthritis, and Hydrops articulorum.

The cervical and lateral ligaments of this joint limit extension and flexion, hence in its luxation, their rupture must take place. Increased thickness of the internal semi-lunar cartilages, produces a deformity which we frequently see, especially among the negro race. It is sometimes so great that when the knees are brought together, the feet are thrown many inches apart; in some only one, in others both joints are thus affected.

The luxations of this joint are complete and incomplete; those which are forward and backward, of the former; those which are internal and external or lateral, of the latter variety. In dislocations of the tibia backwards, there is shortening of the limb; the head of the tibia rests in the popliteal space; immediately above the ligamentum patellæ there is a depression and a corresponding protuberance on the opposite side.

In dislocation of the tibia forwards, the os femoris is thrown backwards; the tibia and patella are drawn forward and upward by the action of the quadriceps muscles. The dislocation backwards and forwards are more generally complete than otherwise. In the internal lateral displacements, the external condyle rests upon the internal fossa of the tibia, and vice versa; the internal or external displacement being indicated by the position of the protuberance. From the anatomy of the knee, we see at once the impossibility of a luxation taking place without a lesion of some of its tissues, especially that of the cervical and lateral ligaments. Owing to the strength of this joint, much force is requsite to displace its bones: so much so, that the injury rarely occurs without the loss of the limb, and the lesion is such as frequently to hazard the life of the patient. When the displacement is complete, as in all ginglymoid joints, very many of the tissues must necessarily be ruptured.

In dislocation of the tibia forwards, the projection of the os femoris backwards may overstretch and even lacerate the popliteal artery.

In all injuries, by displacement of the bones forming a ginglymoid joint, there must be more or less loss of mobility. If the luxation of the joint be complete, then must anchylosis be complete. Although the reduction in this variety of joint be more easily accomplished than any others, our prognosis will always be unfavorable, unless the injury indeed be slight. A partial luxation of the knee joint is not always attended with danger; but if it be at all complicated, fatal consequences may supervene. I remember to have seen but one case reported of recovery from compound luxation of the knee joint. This case was reported by Dr. B. O. Jones, in your Journal of 1855. This case should teach us that all compound dslocations of the knee do not require immediate amputation.

Liston says, "Most frequently fracture is concomitant, perhaps with wound; and such accidents require amputation, either primarily or secondarily." He also says, that under more favorable circumstances, "The joint will remain long weak and never recover entirely." The indications are:

1st. Reduction of dislocation.

2d. Prevent or control inflammation.

3d. Promote the union of torn ligaments.

4th. Prevent anchylosis.

The reduction of the joint should, of course, be accomplished as soon as possible; this may be done generally with little or no difficulty, in consequence of the rupture of the ligamentous structure. If, however, difficulties present themselves from spasmodic action of the muscles, pullies may be requsite, or what is preferable, Dr. Jarvis' adjuster, which affords the means of making the most efficient extension. In luxations of ginglymoid joints, generally, little or no difficulty will attend their reduction; after the replacement, a roller or bandage should be applied to the whole extent of the limb; and if the external injuries would admit, splints should be applied. When the muscles surrounding the joint are lacerated or otherwise injured, the use of splints may be improper; under these circumstances the limb should, after bandaging, be confined to an inclined plane, by bandaging first the foot and then the leg to the plane.

2d indication, will be to control inflammatory action in the joint, which is very important to the patient's recovery. The limb should be kept at perfect rest, while cold applications should be continually made. If general excitement becomes intense, it will be necessary to use antiphlogistic remedies; such as tartarized antimony and laxatives to the bowels. It will also be necessary that the nervous system become not too much excited; that opiates be occasionally administered; venesection may be requisite, but unless imperatively so, should be avoided.

3d indication, is to promote the union of the torn ligaments. In this our efforts will be alone directed to nature's assistance. The limb should be kept at rest, the patient composed, and inflammation subdued; after which it may be necessary to use stimulating applications to the surface of the joint. The adhesion of torn ligaments *must* be a slow process, and many months will be required for their firm adhesion.

4th indication, is to prevent stiffness of the joint: this will rarely be effected. Passive motion commenced, after the subsidence of inflammation, and regularly kept up, affords the only means by which anchylosis may be prevented.

In connection with the preceding remarks, I will mention a case which occured in my practice some two years ago. A gentleman was thrown from his horse while on his way from the plough; his foot became entangled in the harness, and he was dragged a considerable distance, at full speed of the horse. I was called to see him some six or eight hours after the accident, when I found him in a very deplorable condition, with his knee joint completely dislocated.

I could not tell what the direction of the dislocation first was, for so complete was the rupture of the ligaments and cartilages connecting the joint, that I could, with a slight effort, place the joint in any position I desired.

When I first saw him, however, the external condyle of the right knee was resting upon the inner articular surface of the tibia; but I could, with ease, change the position by moving the internal condyle upon the external articular surface.

The forward and backward luxations could, with the same facility, be made. The patella was thrown far back upon the outer surface of the external condyle, its reduction being far more tedious than that of the joint.

Thus we see, there must have been very great laceration of the tissues of the joint; the muscles around the joint were also bruised and lacerated, together with many bruises upon different parts of the body. Under all the circumstances, I was extremely fearful the patient would succumb to the iflammatory excitement which must inevitably ensue. After the reduction of the joint and patella, a roller bandage was applied to the whole extent of the limb, securing the patella in its place by pads.

I was fearful of the application of splints, and used, instead, an inclined plane, (to which the limb was securely bandaged,) the foot, at the same time, being well attached to the foot pieces at the extremity of the plane. After the joint was made comfortable and the face dressed, which was considerably bruised, I commenced at once the antiphlogistic treatment, by first giving him a laxative, and succeeding its action by the use of tartarized antimony and opium. The cold water dressing was continually kept to the joint, by which means it was never allowed to become hot.

In three or four weeks passive motion was commenced, to as great an extent as the pains it produced would allow. I was well satisfied the joint must be stiff, and placed it in that position which was the most convenient. Several months elapsed, and yet the ligaments were not sufficiently united to prevent slight motion upward and downward by pressure upon the joint.

The patient, worn out with his confinement, left his bed, while yet the joint was not firm in its natural position. The consequence was, that the head of the tibia fell a little backward, in which position the limb recovered, leaving a slight deformity; anchylosis was of course complete.

